# Polymorphisms in the *Calcium-Sensing Receptor* Gene Are Associated with Clinical Outcome of Neuroblastoma

**DOI:** 10.1371/journal.pone.0059762

**Published:** 2013-03-22

**Authors:** Laia Masvidal, Raquel Iniesta, Carla Casalà, Patricia Galván, Eva Rodríguez, Cinzia Lavarino, Jaume Mora, Carmen de Torres

**Affiliations:** 1 Developmental Tumor Biology Laboratory, Hospital Sant Joan de Déu and Fundació Sant Joan de Déu, Barcelona, Spain; 2 Unitat de Recerca i Desenvolupament, Parc Sanitari Sant Joan de Déu, Fundació Sant Joan de Déu, Barcelona, Spain; MOE Key Laboratory of Environment and Health, School of Public Health, Tongji Medical College, Huazhong University of Science and Technology, China

## Abstract

**Background:**

Neuroblastic tumors include the neuroblastomas, ganglioneuroblastomas, and ganglioneuromas. Clinical behavior of these developmental malignancies varies from regression to aggressive growth with metastatic dissemination. Several clinical, histological, genetic, and biological features are associated with this diversity of clinical presentations. The calcium-sensing receptor (CaSR) is a G-protein coupled receptor with a key role in calcium homeostasis. We have previously reported that it is expressed in benign, differentiated neuroblastic tumors, but silenced by genetic and epigenetic events in unfavorable neuroblastomas. We have now analyzed three functionally relevant polymorphisms clustered at the signal transduction region of the CaSR (rs1801725, rs1042636 and rs1801726) to assess if genetic variants producing a less active receptor are associated with more aggressive disease course.

**Methods:**

Polymorphisms were analyzed in DNA samples from 65 patients using specific Taqman Genotyping Assays.

**Results:**

Mildly inactivating variant rs1801725 was associated with clinical stage 4 (*P* = 0.002) and the histological subgroup of undifferentiated neuroblastomas (*P* = 0.046). Patients harboring this polymorphism had significantly lower overall (*P* = 0.022) and event-free survival (*P* = 0.01) rates than those who were homozygous for the most common allele among Caucasians. However, this single locus genotype was not independently associated with outcome in multivariate analyses. Conversely, the tri-locus haplotype TAC was independently associated with an increased risk of death in the entire cohort (Hazard Ratio = 2.45; 95% Confidence Interval [1.14–5.29]; *P = *0.022) and also in patients diagnosed with neuroblastomas (Hazard Ratio = 2.74; 95% Confidence Interval [1.20–6.25]; *P* = 0.016).

**Conclusions:**

The TAC haplotype includes the moderately inactivating variant rs1801725 and absence of the gain-of-function rs1042636 polymorphism. Thus, its association with metastatic disease and poor outcome would add to our previous data and further support that inactivation of the *CaSR* gene is a mechanism associated with neuroblastoma malignant behavior.

## Introduction

Neuroblastic tumors are developmental malignancies that arise from peripheral nervous system precursor cells and include the neuroblastomas, ganglioneuroblastomas and ganglioneuromas. Some of these tumors undergo spontaneous regression, others develop as a localized mass and a third group proliferate aggressively and invade organs distant from primary site [1]. A number of clinical, histological, genetic and biological factors are associated with this variety of clinical presentations, including age at diagnosis, clinical stage, histological classification, amplification of the oncogene *MYCN,* alterations of ploidy and abnormalities at the chromosomal region 11q [2]–[4].

The calcium ion (Ca^2+^) plays a key role in a variety of physiological functions including muscle contraction, coagulation and synaptic transmission. In cancer, this divalent cation participates in the control of the balance between cell proliferation and death [5]. One of the mechanisms that contributes to regulate the function of Ca^2+^ in normal and neoplastic cells is the control of its intracellular and plasmatic levels. This is achieved via a complex multiorganic system that involves numerous molecules. Among them, the calcium-sensing receptor (CaSR) is a G-protein coupled receptor (GPCR) that senses minimal plasmatic fluctuations of Ca^2+^ and regulates the secretion of parathyroid hormone and calcitonin accordingly [6]. The CaSR is also expressed in other organs involved in calcium homeostasis, in several non-neoplastic tissues and in a number of malignancies [7], [8]. The role of this GPCR seems to be cell-type dependent, but the mechanisms responsible for this diversity of functions are only partially understood.

The CaSR is composed of three main regions: A large extracellular domain, where the interaction with the Ca^2+^ occurs, a seven transmembrane domain that is a common feature of all GPCRs, and a carboxyl-terminal intracellular tail, necessary for Ca^2+^-mediated activation of G proteins, cell surface expression and phospholipase C activation, among other functions [9]–[11].

Naturally-occurring inactivating mutations of the *CaSR* gene are the cause of familial hypocalciuric hypercalcemia and neonatal severe hyperparathyroidism, while activating mutations produce autosomal dominant hypercalcemia [12]–[16]. Also, more than 450 single nucleotide polymorphisms (SNPs) have been described in the *CaSR* gene. They are divided into three haplotype blocks, coincident with 5′ regulatory, coding and 3′ regulatory domains [17], [18]. Three nonsynonymous variants at the intracellular tail encoded by exon 7 have been identified. The A986S change encoded by the most common SNP of the *CaSR* gene in Caucasians, c.2956 G>T (rs1801725), is considered to produce a moderately less active receptor [18], [19]. The glycine substitution at arginine-990 encoded by c.2968 A>G (rs1042636) might induce gain of function [20]. Finally, c.3031 C>G, or Q1011E (rs1801726), is a common polymorphism in populations with African ancestry whose functional significance remains to be established [16], [18]. Besides their respective function, specific haplotypes in this cluster of polymorphisms have been found in association with blood ionized calcium levels, primary hyperparathyroidism and nephrolitiasis [21]–[23].

Several studies have analyzed the association of these genetic variants of the CaSR with risk of colorectal or prostate cancer [24]–[30]. However, to the best of our knowledge, a specific haplotype at these three loci has never been reported as a potential predictor of outcome in any malignancy.

Our group reported for the first time the expression of the CaSR in a developmental malignancy. We found that it is expressed in differentiated, favorable neuroblastic tumors, and up-regulated upon differentiation induction [31]. Recently, we have also shown that the *CaSR* gene is silenced by genetic and epigenetic mechanisms in unfavorable neuroblastomas [32]. To gain further insight into the genetic mechanisms responsible for its inactivation in malignant neuroblastomas, we have now analyzed the three clustered genetic variants at coding exon 7 that have been described to influence CaSR activity. Based on our prior data, we hypothesized that allelic variants encoding a less active receptor might be associated with poor clinical outcome.

## Materials and Methods

### Ethics Statement

Written informed consent was obtained from patients, parents or legal guardians. The protocol was in accordance with the Declaration of Helsinki and approved by the Ethics Committee for Clinical Research (Comité Ético de Investigación Clínica, Fundació Sant Joan de Déu, Esplugues de Llobregat, Barcelona).

### Patients and Samples

Peripheral blood and bone marrow samples (n = 65) were collected from all patients newly diagnosed with neuroblastic tumors at Hospital Sant Joan de Déu, Barcelona, Spain, with a follow-up time ≥12 months. Bone marrow samples were only included in the study if they were free of neuroblastoma cells, as assessed by *PHOX2B* mRNA expression analysis (see below). Age at diagnosis, clinical stage (International Neuroblastoma Staging System), *MYCN* amplification status, International Neuroblastoma Pathology Classification (INPC), ethnicity and time to follow-up were recorded.

Patients were uniformly treated according to the following criteria: Stage 4s cases received no cytotoxic therapy unless life-threatening condition occurred during observation; *MYCN* non-amplified stages 1, 2 and 3 tumors were managed exclusively with surgery unless clinically relevant progression occurred; and stage 4 and *MYCN*-amplified cases received induction chemotherapy, second-look surgery, radiotherapy, myeloablative therapy followed by autologous bone marrow transplant, anti-GD2 immunotherapy and retinoic acid.

### *PHOX2B* mRNA Expression Analysis

Bone marrow mononuclear cell isolation, total RNA extraction and cDNA synthesis were carried out as described [33]. Analysis of *PHOX2B* mRNA expression was performed following previously reported procedures [34] in a 7000 Sequence Detection System (Applied Biosystems). Only samples with a Ct for reference gene <30 were considered of good quality and then evaluated. Samples with any amplification of the neuroblastoma-specific *PHOX2B* marker were scored as positive and thus matching DNA was excluded from genotyping analysis.

### Genotyping

Genomic DNA was isolated from peripheral blood (n = 41) or bone marrow (n = 24) mononuclear cells using Qiagen reagents (Qiagen, Valencia, CA). Polymorphisms were analyzed in a 7500 real-time PCR system (Applied Biosystems) using specific SNP Genotyping Assays (Applied Biosystems references 7504853 for rs1801725, 7504854 for rs1042636 and 7504855 for rs1801726), following manufacturer’s instructions. Genotyping (L.M.), clinical data recording (C.C.) and statistical analyses (R.I.) were performed by three independent researchers. For quality control, random cases (15%) were processed twice in separate runs. Results were 100% concordant.

### Statistical Analyses

The χ^2^ and Fisher’s exact tests were used to assess the association of specific genotypes with clinical and biological subgroups. Elapsed time from time of diagnosis to an event (relapse, progression or death) or the end of follow-up was used to compute event-free survival (EFS) and overall survival (OS) probabilities, according to the method of Kaplan and Meier [35]. The log-rank statistic was used to compare the EFS and OS probabilities between groups [36]. The prognostic significance of variables was assessed by Cox proportional models [37]. SNPStats web tool was used to analyze individual SNPs [38]. Homozygous cases for the most frequent allele were taken as the reference level, and heterozygous and homozygous cases for the minor allele were grouped. THESIAS (Testing Haplotype EffectS In Association Studies) program and BayHap R package were used to compare OS and EFS probabilities among different haplotype blocks [39]–[41]. Haplotype frequencies were estimated in the total cohort, and separately in the subgroups of alive and dead patients, event and event-free subgroups. Departure form Hardy-Weinberg equilibrium was assessed by means of the Fisher’s exact. Linkage disequilibrium (LD) between polymorphisms was measured through *D′* and *r^2^*. In Cox regression analysis, the most common haplotype in the sample was taken as the reference level. All models were adjusted for age at diagnosis, clinical stage and *MYCN* amplification status. Additivity of haplotype effects was considered in all models after performing Likelihood Ratio tests. Analyses were performed with R 2.14.1, SNPstats or THESIAS programs. *P*<0.05 was considered significant. Estimates of EFS and OS and hazard ratio (HR) are presented with 95% confidence intervals (CI).

This study was conceived to address a single hypothesis, i.e. that functionally relevant polymorphisms of the *CaSR* gene influence neuroblastoma phenotype and thus survival probabilities of patients. This single hypothesis was tested and proved by means of a single model. Therefore, results were interpreted without adjusting for multiple tests [42]. However, the potential clinical relevance of exploring the entire cohort of patients diagnosed with neuroblastic tumors and those neuroblastomas without amplification of the oncogene *MYCN*, prompted us to describe survival probabilities in these patients as well.

## Results

### *CaSR* Gene Exon 7 Polymorphisms in Neuroblastic Tumors

All available patients diagnosed with neuroblastic tumors with a follow-up time longer than 12 months were included in the study (n = 65). Their clinical and biological features are summarized in Table S1 and detailed in Table S2. Median follow-up was 4.4 years (range 12.2 to 124.3 months). All patients but one (#61) were of Caucasian origin. To ascertain if this cohort was similar to others, OS and EFS probabilities were calculated according to well-established clinical and biological parameters in neuroblastoma (Table S1). As expected, clinical stage 4, *MYCN* amplification and unfavorable histology were associated with poor outcome. Age at diagnosis ≥18 months was correlated with worse OS (*P* = 0.013) and EFS (*P* = 0.001) rates among neuroblastoma patients but not in the entire cohort, due to the presence of ganglioneuroblastomas and ganglioneuromas (Table S1 and Table S2).

Statistical associations of single locus genotypes were then examined. Polymorphism rs1801725, present either in homo- or heterozygosis, was detected in 22/65 (33.8%) of patients. Genetic variant rs1042636 was found in 8/65 patients (12.3%) and rs1801726 was only detected in four patients (6.1%) (Table S2). The genotype frequencies for all three polymorphisms were in accordance with Hardy-Weinberg equilibrium (Table S3). Associations of single SNPs with clinical and biological features of neuroblastic tumors are shown in Table 1. The low frequency of two variants, rs1042636 and rs1801726, precluded the analysis of statistical correlations with specific subgroups or survival probabilities. Conversely, upon univariate analysis, rs1801725 was found to be associated with clinical stage 4 (*P* = 0.002) and the histological subgroup of undifferentiated neuroblastomas (*P* = 0.046). Patients harboring this variant had significantly lower OS (*P* = 0.022) and EFS (*P* = 0.01) rates than those who were homozygous for the most common allele among Caucasians. However, this single locus genotype was not independently associated with outcome in multivariate analyses (Table 2).

**Table 1:** Association of *CaSR* gene polymorphisms and haplotypes with clinical and biological features of neuroblastic tumors.

	Age*		INSS		*MYCN* status		INPC	
	<18	≥18	1,2,3,4s	4	NA	A	Favorable	Unfavorable
**rs1801725**								
G/G	16	27	29	14	34	9	25	18
G/T+T/T	8	14	6	16	17	5	8	14
* P*	0.947		0.002		0.868		0.096	
**rs1042636**								
A/A	22	36	32	26	44	14	29	29
A/G+G/G	2	5	3	4	7	0	4	3
* P*	0.628		0.537		0.142		0.721	
**rs1801726**								
C/C	22	39	33	28	13	48	30	31
C/G	2	2	2	2	1	3	3	1
* P*	0.576		0.873		0.862		0.317	
**Haplotypes**								
G-A-C	33	60	58	35	21	72	31	62
T-A-C	10	15	6	19	6	19	12	13
G-G-C	3	5	4	4	0	8	0	8
G-A-G	2	2	2	2	1	3	1	3
* P*	0.923		0.008		0.499		0.090	

* Age at diagnosis: months. INSS: International Neuroblastoma Staging System. *MYCN* status: A - amplified; NA - not amplified. INPC: International Neuroblastoma Pathology Classification.

**Table 2:** Overall and event-free survival of patients diagnosed with neuroblastic tumors according to *CaSR* genotypes.

		OS				EFS			
Genotype	Cases (n = 65)	Deaths (n = 20)	Log-rank *P*	HR^†^ (95% CI)	*P* ‡	Events (n = 25)	Log-rank *P*	HR^†^ (95% CI)	*P* ‡
**rs1801725**									
G/G	43	9	0.022	1.0	0.183	12	0.01	1.0	0.084
G/T+T/T	22	11		2.217 (0.686–7.158)		13		2.218 (0.114–0.714)	
**rs1042636**									
A/A	58	18	0.808	1.0	0.812	23	0.67	1.0	0.831
A/G+G/G	7	2		1.207 (0.255–5.705)		2		0.850 (0.191–3.790)	
**rs1801726**									
C/C	61	19	0.99	1.0	0.83	24	0.735	1.0	0.696
C/G	4	1		0.797 (0.101–6.285)		1		0.669 (0.089–5.035)	

OS: Overall survival. EFS: Event-free survival.

HR^†^: Hazard ratio.

‡ *P* of Hazard Ratio. CI: Confidence interval.

Multivariate Cox regression model with adjustment for age at diagnosis, clinical stage and *MYCN* amplification status.

Associations of tri-locus haplotypes were then evaluated. Four haplotypes were found in the entire cohort (Table 3). The most probable haplotype (GAC) had an estimated frequency of 72% and was composed of the most frequent alleles among Caucasian populations, as expected. The estimated frequency of the TAC haplotype was nearly 20% and lower for two other combinations (GGC and GAG). Strong LD (*D*′>0.95) existed between all SNP pairs with the only exception of rs1801725 and rs1801726 (Table S4). This result might be influenced by the low frequence of rs1801726 minor allele in populations of Caucasian origin.

**Table 3:** Overall and event-free survival of patients diagnosed with neuroblastic tumors according to *CaSR* haplotypes.

		OS				EFS			
Haplotype*	Total Sample** (n = 65)	Alive(n = 45)	Dead (n = 20)	HR^†^ (95% CI)	*P* ‡	Event-free (n = 40)	Event (n = 25)	HR (95% CI)	*P* ‡
G-A-C	72	77	60	1.00		76	64	1.00	
T-A-C	19	13	32	2.45 (1.14–5.29)	0.022	13	30	2.02 (0.99–4.12)	0.052
G-G-C	6	7	5	1.14 (0.22–5.80)	0.868	4	2	0.57 (0.06–5.01)	0.616
G-A-G	3	3	3	0.59 (0.07–5.19)	0.636	8	4	0.81 (0.18–3.71)	0.786

* rs1801725-rs1042636-rs1801726.

** Haplotype estimated frequencies (%). OS: Overall survival. EFS: Event-free survival.

HR^†^Hazard ratio.

‡ *P* of Hazard Ratio. CI: Confidence interval. Multivariate Cox regression model with adjustment for age at diagnosis, clinical stage and *MYCN* amplification status.

**Figure 1:**
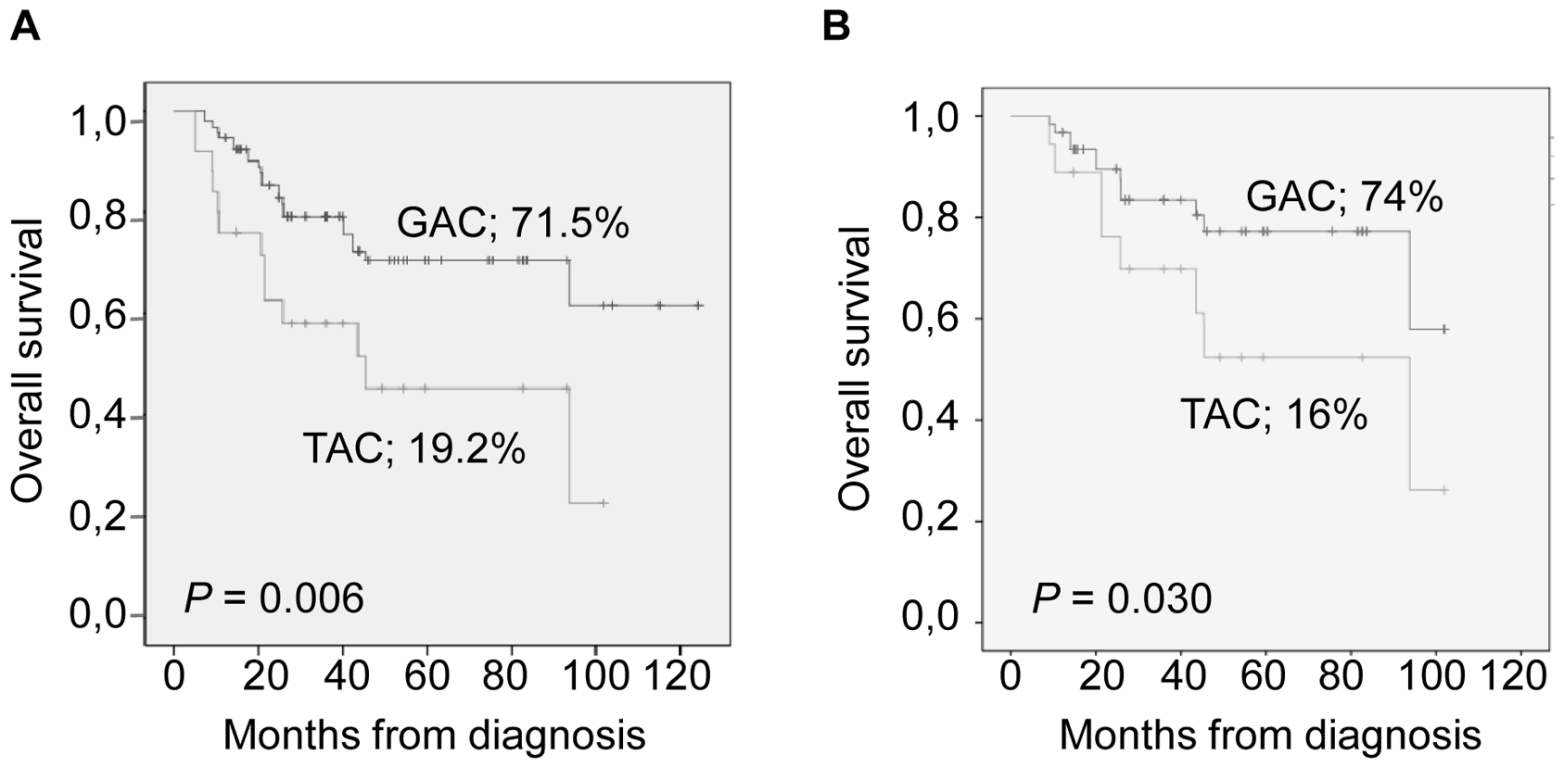
Overall survival probabilities according to *CaSR* gene haplotypes. Overall survival probabilities of patients diagnosed with (A) neuroblastic tumors or (B) neuroblastomas according to *CaSR* gene haplotype at loci rs1801725, rs1042636 and rs1801726.

In univariate analysis, the TAC haplotype was correlated with clinical stage 4 (*P* = 0.008) and inferior OS rates (*P* = 0.006) (Figure 1A). Moreover, in multivariate analyses, the TAC combination was independently associated with an increased risk of death in the entire cohort after adjusting for age at diagnosis, clinical stage and *MYCN* amplification status (Table 3). This association was also significant when the analysis was restricted to patients with a follow-up time longer than three years (HR = 2.19; 95% CI [1.01–4.76]; *P* = 0.046). An increased risk of events (*P* = 0.008) that almost reached significance in multivariate analyses (*P = *0.052) was also observed among patients with the TAC haplotype (Table 3). Associations of this combination when present in homozygosis were not analyzed separately due to their very low frequency.

### *CaSR* Gene Exon 7 Polymorphisms in Neuroblastoma

The subgroup of neuroblastoma patients (n = 54) was then analyzed separately according to single locus genotypes and tri-locus haplotypes. Associations of single SNPs with clinical and biological features of neuroblastomas are shown in Table S5. Again, rs1801725 variant, present either in homo- or heterozygosis, was associated with clinical stage 4 (*P* = 0.02) and inferior OS rates (*P* = 0.023). However, this polymorphism was not an independent predictor of outcome in multivariate analyses, after adjusting for age at diagnosis, clinical stage and *MYCN* status. The other two SNPs were not statistically associated with any of the prognostic factors examined or clinical outcome (Table S5).

When tri-locus combinations were assessed, estimated frequencies of haplotypes were similar to the ones found in the entire cohort (Table 4). Strong LD (*D*′>0.95) was found between all SNP pairs with the only exception of rs1801725 and rs1801726 (Table S4). In this sample set, the TAC haplotype was also correlated with clinical stage 4 (*P* = 0.012) and inferior OS rates (*P* = 0.03) (Table S5 and Figure 1B). Moreover, it was independently associated with an increased risk of death (Table 4), and this correlation was still observed among neuroblastoma patients with a follow-up time longer than three years (HR = 2.38; 95% CI [1.04–5.45]; *P* = 0.039). An increased risk of events was also seen among neuroblastoma patients with the TAC haplotype (*P* = 0.035), although this association was not statistically significant in multivariate analyses (Table 4).

**Table 4:** Overall and event-free survival of patients diagnosed with neuroblastomas according to *CaSR* haplotypes.

		OS				EFS			
Haplotype*	Total Sample** (n = 54)	Alive (n = 35)	Dead (n = 19)	HR^†^ (95% CI)	*P* ‡	Event- free (n = 30)	Event (n = 24)	HR^†^ (95% CI)	*P* ‡
G-A-C	69	74	58	1.00		73	63	1.00	
T-A-C	22	16	34	2.74 (1.20–6.25)	0.016	15	31	1.95 (0.94–4.04)	0.072
G-G-C	6	7	5	1.29 (0.24–6.87)	0.763	8	4	0.82 (0.178–3.85)	0.809
G-A-G	3	3	3	0.74 (0.08–6.71)	0.790	3	2	0.64 (0.07–5.63)	0.689

* rs1801725-rs1042636-rs1801726.

** Haplotype estimated frequencies (%). OS: Overall survival. EFS: Event-free survival.

HR^†^Hazard ratio.

‡ *P* of Hazard Ratio. CI: Confidence interval. Multivariate Cox regression model with adjustment for age at diagnosis, clinical stage and *MYCN* amplification status.

Among the subgroup of neuroblastomas without amplification of the *MYCN* oncogene (n = 40), the TAC combination was correlated with inferior EFS rates (*P* = 0.037). The association with lower OS rates did not reached statistical significance (*P = *0.066). In multivariate analyses, the TAC haplotype was not an independent predictor of outcome in this subgroup of neuroblastomas, after adjusting for age at diagnosis, clinical stage and *MYCN* amplification status.

## Discussion

Over the last decades, somatic alterations associated with different neuroblastoma phenotypes have been extensively analyzed. More recently, several genome-wide association studies have identified susceptibility loci and alleles associated with low- or high-risk disease. They include variations at *FLJ22536* (6p22), *BARD1* (2q35), *LMO1* (11p15), *HSD17B12* (11p11.2), *DUSP12* (1q23.3), *LINC00340* (6p22), *HACE1* and *LIN28B* (6q16) [43]–[49]. These accumulating data would support the hypothesis that a combination of variants might contribute to the malignant transformation of embryonal precursor cells from which neuroblastic tumors originate, and influence their phenotype. Thus, benign and malignant forms of neuroblastic tumors might arise as a result of different initiating genetic events [44], [49]. Our present results would add to this growing body of evidence and further support that genetic variations play a fundamental role in these developmental malignancies.

We have previously reported that the *CaSR* gene is expressed in differentiated, benign neuroblastic tumors but silenced by genetic and epigenetic events in undifferentiated, unfavorable neuroblastomas. Moreover, upon ectopic overexpression and reactivation of the CaSR, neuroblastoma cells undergo apoptosis [31]–[32]. Altogether, these data were consistent with the hypothesis that the CaSR exerts tumor-suppressor functions in the context of neuroblastic tumors. To gain further insight into the genetic mechanisms responsible for its inactivation in aggressive neuroblastomas, we have now analyzed three functionally relevant genetic variants clustered at the carboxyl-terminal tail of the receptor. They have been analyzed in the context of other neoplasias mostly as susceptibility loci [24]–[30]. However, based on our previous results, we hypothesized that their ability to modify CaSR function might influence neuroblastoma phenotype and thus clinical outcome.

The cohort analyzed was almost uniformly composed of patients of Caucasian origin and therefore the most frequent variant was rs1801725. The frequency of the two other polymorphisms was not significantly different from other Caucasian populations [50]. Minor allele rs1801725 is thought to moderately reduce CaSR activity [18], [19]. In keeping with our previous data, this allegedly less active form of the CaSR was associated with the histological subgroup of undifferentiated neuroblastomas, metastatic disease and inferior OS rates. However, it was not an independent predictor of outcome in multivariate analyses.

Interestingly, when the three polymorphisms were analyzed as a block, the tri-locus haplotype TAC was correlated with clinical stage 4 and independently associated with an increased risk of death in the entire cohort of neuroblastic tumors and, more importantly, among patients diagnosed with neuroblastomas. Although the cohort examined is not large, patients have been diagnosed and treated at a single institution according to homogeneous criteria. Thus, their outcome is more likely to depend on clinical features and tumor biology than on management. Moreover, the association of this haplotype with poor outcome was also statistically significant among patients with a longer follow-up time. However, the frequency of two variants, rs1042636 and rs1801726, was quite low and limited the statistical power to detect associations. Thus, validation of our findings in larger, independent cohorts is warranted. Furthermore, analysis of patients from different population backgrounds will be necessary to determine if this haplotype is associated with neuroblastoma phenotype and outcome in patients of other ethnic origins.

These three SNPs were first suggested to act as a cluster in 1996 [21]. Linkage disequilibrium between them has been repeatedly reported, a fact that might interfere in the evaluation of specific functional effects derived from each polymorphism [18]. Scillitani and colleagues described specific haplotypes at these three loci in association with blood ionized calcium levels and nephrolitiasis [22], [23]. Functional *in* *vitro* analyses to understand the biological significance of the different haplotypes are lacking. However, it is tempting to speculate that the TAC combination might encode a moderately less active form of the CaSR due to the presence of rs1801725 and the absence of the gain of function produced by rs1042636. In the context of neuroblastic tumors, the association of this potentially less active CaSR with metastatic disease and poor outcome would add to our previous results indicating that this gene is silenced by genetic and epigenetic mechanisms in aggressive neuroblastomas.

Several independent predictors of outcome in neuroblastoma have been reported by other groups and ours [2]–[4], [51]–[54]. However, high-quality tumor samples and laborious, expensive techniques are required to analyze most of these genetic and biological features. Conversely, the analysis of polymorphisms can be carried out in DNA isolated from any patient’s sample, an advantage when a biopsy is not feasible, or the quality or quantity of the available primary tumor is sub-optimal. Moreover, it is simple to perform and to interpret, highly reproducible and cost-effective.

In summary, our present data indicate that a tri-locus haplotype at the signal transduction region of the CaSR might be an independent predictor of outcome in patients diagnosed with neuroblastic tumors. Moreover, we provide new evidence supporting that inactivation of the *CaSR* gene is a mechanism associated with neuroblastoma malignant behavior.

## Supporting Information

Table S1 Overall and event-free survival of patients diagnosed with neuroblastic tumors according to clinical and biological features. Univariate and multivariate analyses were conducted to analyze overall and event-free survival probabilities in the entire cohort of patients diagnosed with neuroblastic tumors (n = 65) according to well-established prognostic factors in these malignancies.
(DOCX)

Table S2 Clinical, histological and biological features of patients diagnosed with neuroblastic tumors and their genotype at three polymorphisms of the *CaSR* gene. The following data are provided for each patient in the cohort: clinical data at diagnosis (age in months and clinical stage according to the International Neuroblastoma Staging System), follow-up data required to calculate event-free and overall survival probabilities, histological subgroup, *MYCN* amplification status and genotype at three *CaSR* gene polymorphisms (rs1801725, rs1042636 and rs1801726).
(XLSX)

Table S3 Analysis of departure from Hardy-Weinberg equilibrium. Fisher’s exact text was carried out in the entire cohort of patients diagnosed with neuroblastic tumors (n = 65) and in those with neuroblastomas (n = 54) to assess if genotype frequencies at polymorphisms rs1801725, rs1042636 and rs1801726 of the *CaSR* gene were in accordance with Hardy-Weinberg equilibrium.
(DOCX)

Table S4 Linkage disequilibrium between loci rs1801725, rs1042636 and rs1801726 in patients diagnosed with (A) neuroblastic tumors and (B) neuroblastomas. Linkage disequilibrium between polymorphisms pairs was measured through *D′* and *r^2^*. These calculations were conducted in the entire cohort of neuroblastic tumors (A) and in patients diagnosed with neuroblastomas (B).
(XLSX)

Table S5 Association of *CaSR* gene polymorphisms and haplotypes with clinical and biological features of neuroblastomas. Statistical analyses were conducted in neuroblastoma patients (n = 54) to evaluate associations between single locus genotypes or tri-locus haplotypes at three *CaSR* gene polymorphisms (rs1801725, rs1042636 and rs1801726) and independent prognostic factors in these malignancies (age at diagnosis, clinical stage, *MYCN* amplification status and histological subgroup according to Shimada classification).
(DOCX)
