# Supplementary material for: Polymorphisms in the Calcium-Sensing Receptor Gene Are Associated with Clinical Outcome of Neuroblastoma
Source: PLoS One. 2013 Mar 22;8(3):e59762. doi: 10.1371/journal.pone.0059762 (PMC3606108; doi:10.1371/journal.pone.0059762)
Supplement: Table S1 — Overall and event-free survival of patients diagnosed with neuroblastic tumors according to clinical and biological features. Univariate and multivariate analyses were conducted to analyze overall and event-free survival probabilities in the entire cohort of patients diagnosed with neuroblastic tumors (n = 65) according to well-established prognostic factors in these malignancies. (DOCX) [file pone.0059762.s001.docx]

**Table S1.** Overall and event-free survival of patients diagnosed with neuroblastic tumors according to clinical and biological features.

|  |  | OS | | | |  | EFS | | | |
| --- | --- | --- | --- | --- | --- | --- | --- | --- | --- | --- |
| Variables | Cases (n=65) | Deaths (n=20) | Log-rank *P* | HR† (95% CI) | *P*‡ |  | Events (n=25) | Log-rank *P* | HR (95% CI) | *P*‡ |
| Age* |  |  |  |  |  |  |  |  |  |  |
| <18 | 24 | 4 | 0.073 | 1 | 0.895 |  | 4 | 0.009 | 1 | 0.294 |
| ≥ 18 | 41 | 16 |  | 1.079 (0.349-3.334) |  |  | 21 |  | 0.554 (0.184-1.671) |  |
| INSS |  |  |  |  |  |  |  |  |  |  |
| 1,2,3,4s | 35 | 2 | <0.001 | 1 | <0.001 |  | 5 | <0.001 | 1 | <0.001 |
| 4 | 30 | 18 |  | 20.108 (4.341-93.138) |  |  | 20 |  | 6.868 (2.365-19.944) |  |
| *MYCN* status |  |  |  |  |  |  |  |  |  |  |
| Not amplified | 51 | 11 | <0.001 | 1 | 0.006 |  | 15 | 0.001 | 1 | 0.037 |
| Amplified | 14 | 9 |  | 3.921 (1.487-10 338) |  |  | 10 |  | 2.384 (1.056-5.385) |  |
| INPC |  |  |  |  |  |  |  |  |  |  |
| Favorable | 33 | 0 | -- | -- | -- |  | 0 | -- | -- | -- |
| Unfavorable | 32 | 20 |  | -- |  |  | 25 |  | -- |  |

*Age at diagnosis (months). INSS: International Neuroblastoma Staging system. INPC: International Neuroblastoma Pathology Classification. OS: Overall survival. EFS: Event-free survival. HR†: Hazard ratio. ‡: *P* of Hazard Ratio. CI: Confidence interval. Multivariate Cox regression model with adjustment for the indicated variables.
