# Supplementary material for: Polymorphisms in the Calcium-Sensing Receptor Gene Are Associated with Clinical Outcome of Neuroblastoma
Source: PLoS One. 2013 Mar 22;8(3):e59762. doi: 10.1371/journal.pone.0059762 (PMC3606108; doi:10.1371/journal.pone.0059762)
Supplement: Table S3 — Analysis of departure from Hardy-Weinberg equilibrium. Fisher’s exact text was carried out in the entire cohort of patients diagnosed with neuroblastic tumors (n = 65) and in those with neuroblastomas (n = 54) to assess if genotype frequencies at polymorphisms rs1801725, rs1042636 and rs1801726 of the CaSR gene were in accordance with Hardy-Weinberg equilibrium. (DOCX) [file pone.0059762.s003.docx]

| **Table S3.** Analysis of departure from Hardy-Weinberg equilibrium. |
| --- |
|  |

| N | SNP | Total sample |
| --- | --- | --- |
|  | rs1801725 | 0.69 |
| 65 | rs1042636 | 0.2 |
|  | rs1801726 | 1 |
|  | rs1801725 | 0.71 |
| 54 | rs1042636 | 0.19 |
|  | rs1801726 | 1 |
