# Supplementary material for: Polymorphisms in the Calcium-Sensing Receptor Gene Are Associated with Clinical Outcome of Neuroblastoma
Source: PLoS One. 2013 Mar 22;8(3):e59762. doi: 10.1371/journal.pone.0059762 (PMC3606108; doi:10.1371/journal.pone.0059762)
Supplement: Table S5 — Association of CaSR gene polymorphisms and haplotypes with clinical and biological features of neuroblastomas. Statistical analyses were conducted in neuroblastoma patients (n = 54) to evaluate associations between single locus genotypes or tri-locus haplotypes at three CaSR gene polymorphisms (rs1801725, rs1042636 and rs1801726) and independent prognostic factors in these malignancies (age at diagnosis, clinical stage, MYCN amplification status and histological subgroup according to Shimada classification). (DOCX) [file pone.0059762.s005.docx]

**Table S5.**  Association of *CaSR* gene polymorphisms and haplotypes with clinical and biological features of neuroblastomas.

|  | Age* | |  | INSS | |  | *MYCN* status | |  | INPC | |
| --- | --- | --- | --- | --- | --- | --- | --- | --- | --- | --- | --- |
|  | <18 | ≥18 |  | 1,2,3,4s | 4 |  | NA | A |  | Favorable | Unfavorable |
| rs1801725 |  |  |  |  |  |  |  |  |  |  |  |
| G/G | 15 | 18 |  | 20 | 13 |  | 24 | 9 |  | 16 | 17 |
| G/T+T/T | 8 | 13 |  | 5 | 16 |  | 16 | 5 |  | 7 | 14 |
| *P* | 0.594 |  |  | 0.02 |  |  | 0.777 |  |  | 0.272 |  |
| rs1042636 |  |  |  |  |  |  |  |  |  |  |  |
| A/A | 21 | 27 |  | 23 | 25 |  | 34 | 14 |  | 20 | 28 |
| A/G+G/G | 2 | 4 |  | 2 | 4 |  | 6 | 0 |  | 3 | 3 |
| *P* | 0.627 |  |  | 0.499 |  |  | 0.321 |  |  | 0.697 |  |
| rs1801726 |  |  |  |  |  |  |  |  |  |  |  |
| C/C | 22 | 29 |  | 24 | 27 |  | 38 | 13 |  | 21 | 30 |
| C/G | 1 | 2 |  | 1 | 2 |  | 2 | 1 |  | 2 | 1 |
| *P* | 0.739 |  |  | 0.643 |  |  | 0.763 |  |  | 0.386 |  |
| Haplotypes |  |  |  |  |  |  |  |  |  |  |  |
| G-A-C | 32 | 42 |  | 41 | 33 |  | 53 | 21 |  | 31 | 43 |
| T-A-C | 10 | 14 |  | 5 | 19 |  | 18 | 6 |  | 12 | 12 |
| G-G-C | 3 | 4 |  | 3 | 4 |  | 7 | 0 |  | 0 | 7 |
| G-A-G | 1 | 2 |  | 1 | 2 |  | 2 | 1 |  | 1 | 2 |
| *P* | 0.988 |  |  | 0.012 |  |  | 0.427 |  |  | 0.123 |  |

* Age at diagnosis: months. INSS: International Neuroblastoma Staging System. *MYCN* status: A - amplified; NA - not amplified. INPC: International Neuroblastoma Pathology Classification.
